# Phytochemicals Targeting VEGF and VEGF-Related Multifactors as Anticancer Therapy

**DOI:** 10.3390/jcm8030350

**Published:** 2019-03-12

**Authors:** Amna Parveen, Lalita Subedi, Heung Wan Kim, Zahra Khan, Zahra Zahra, Muhammad Qudratullah Farooqi, Sun Yeou Kim

**Affiliations:** 1 Department of Pharmacognosy, Faculty of Pharmaceutical Science, Government College University, Faisalabad, Faisalabad 38000, Pakistan; 2 College of Pharmacy, Gachon University, No. 191, Hambakmoero, Yeonsu-gu, Incheon 21936, Korea; subedilali@gmail.com (L.S.); ettienne1226@gmail.com (H.W.K.); zahra.khan37@gmail.com (Z.K.); 3 Institute of Environmental Sciences and Engineering, School of Civil and Environmental Engineering, National University of Sciences and Technology, Sector H-12, Islamabad 44000, Pakistan; zahra@iese.nust.edu.pk; 4 School of Agriculture and Environment, The University of Western Australia, Perth, WA 6009, Australia; mqfarooqi@gmail.com; 5 Gachon Institute of Pharmaceutical Science, Gachon University, No. 191 Hambakmoe-ro, Yeonsu-gu, Incheon 21936, Korea

**Keywords:** vascular endothelial growth factor, receptor tyrosine kinases, neuropilins, epithelial–mesenchymal transition, hypoxia-inducible factor, estrogen receptor-β, prolyl hydroxylase 2, Notch ligand Delta-like 4, focal adhesion kinases, placental growth factor-1

## Abstract

The role of vascular endothelial growth factor (VEGF) in cancer cells is not limited to angiogenesis; there are also multiple factors, such as neuropilins (non-tyrosine kinases receptors), tyrosine kinases receptors, immunodeficiencies, and integrins, that interact with VEGF signaling and cause cancer initiation. By combating these factors, tumor progression can be inhibited or limited. Natural products are sources of several bioactive phytochemicals that can interact with VEGF-promoting factors and inhibit them through various signaling pathways, thereby inhibiting cancer growth. This review provides a deeper understanding of the relation and interaction of VEGF with cancer-promoting factors and phytochemicals in order to develop multi-targeted cancer prevention and treatment.

## 1. Introduction

Cancer cells have the ability to spread to adjacent organs by a phenomenon known as metastasis [1]. Owing to this ability of cancer cells, a life-threatening condition develops [2]. In the metastasis process, the growth of the vascular network plays a very important role. New blood vessels and lymphatic vessels are formed by mechanisms known as angiogenesis and lymphangiogenesis, respectively [3]. Tumor growth depends on these mechanisms, which are triggered by various signals produced by tumor cells [4]. Tumor cells contain various angiogenic proteins and growth factors, such as vascular endothelial growth factor (VEGF), neuropilins (NRPs), and integrins, that initiate chemical stimulation in the angiogenesis process [5]. VEGF acts as a key regulator of normal and abnormal angiogenesis.

VEGF, a signal protein, is known as a vascular permeability factor. It is identified as an endothelial cell-specific mitogen. The VEGF family contains seven members, namely VEGFA, VEGFB, VEGFC, VEGFD, VEGFE, VEGFF, and placental growth factors (PlGFs). All members of the VEGF family contain a common VEGF homology domain [6]. However, these members are different in terms of their expression pattern, receptor attachment, and biological activities. VEGFA has more similarity with VEGF. VEGFA has several variants, such as VEGF 121, VEGF 145, and VEGF 206. The occurrence of different variants represents alternative splicing receptor specificity and biological functions. VEGF ligands show their effect by binding to different tyrosine kinases and non-tyrosine kinase receptors involved in cancer development. By suppressing or inhibiting these factors through VEGF inhibition, a potential anticancer therapy can be developed. The Food and Drug Administration (FDA) recently approved a humanized anti-VEGF monoclonal antibody, named bevacizumab, as a first-line therapy in combination with chemotherapeutic agents to treat metastatic colorectal cancer [7,8]. However, more effort should be made to achieve targeted delivery therapy.

To achieve targeted delivery therapy, we need to discover compounds or compound mixtures that can interact with several cancer-causing factors through different signaling pathways at the same time. Moreover, finding multi-target strategies to combat aging, cancer, neurodegenerative diseases, etc. through machine learning methods could also be a novel source of potential agents [9,10,11,12]. Interestingly, extracts of natural products or their derived phytochemicals can target and attack several cancer-causing factors, and they are more effective, cheaper, easy to access, and have a low number of side effects, thereby enhancing patient compliance and ultimately being beneficial to achieve a healthy and happy life [13,14,15,16]. This review aims to understand the mechanism underlying VEGF interaction with cancer-causing factors and how they are interlinked with the bioactive constituents that have recently been identified and derived from natural products to inhibit cancer development and metastasis.

## 2. Materials and Methods

An extensive study was carried out to collect the recently published data in various electronic databases, including PubMed, Science Direct, Embase, and Google Scholar. We explored medicinal plants and their potential constituents targeting VEGF and VEGF-associated signaling networks as an anticancer therapy.

## 3. Results

### 3.1. Interaction of VEGF with Cancer-Causing Factors

#### 3.1.1. VEGF Interaction with Immune Cells

The development and survival of cancer cells are multifactor processes that involve genetic mutation and physiological changes within both cancer cells and the body’s defense mechanism. The body’s defense mechanism or immune system plays a supporting role against cancer progression [17,18]. The development of cancer can be controlled or terminated by a process known as immune surveillance. Dendritic cells (DCs) are the most potent antigen-presenting cells that play a central role in anticancer immunity [19]. Tumor cells are capable of developing mechanisms to avoid recognition, control, and inhibition by anticancer immune responses [20]. Owing to the production of soluble factors in cancer, such as interleukin-10 (IL-10), tumor necrotic factor (TNF), transforming growth factor (TGF)-β, and VEGF, the function and action of immune cells are blocked [21]. Overproduction of VEGF in cancer is associated with a poor prognosis. Stimulation or proliferation of endothelial cells is induced by VEGF, which in turn plays a central role in the development of tumor neovasculature [21].

VEGF production from breast and colon cancer cells can dramatically inhibit the differentiation and maturation of DCs from their precursor, a cluster of differentiation (CD)34^+^ [22]. In the presence of VEGF, the DCs produced from stem cells lack normal morphological characteristics and express a low level of major histocompatibility complex (MHC) II [18]. The cells also have a decreased ability to take up soluble antigens. The reason can be explained as follows: in CD34^+^ hematopoietic progenitor cells (HPCs), VEGF binds to the Flt-1 receptor. These HPCs, after being converted to mature DCs, downregulate the receptor, inhibiting its binding with VEGF. Signal transduction between the TNF-α/TNF-α receptors on the cell surface and the nuclear factor kappa beta (NF-kB)/inhibitory kB complex in the nucleus is responsible for the differentiation of CD34^+^HPCs into DCs [23]. On the contrary, VEFG inhibits NF-kB activation, which ultimately prevents DC differentiation. When DC development is blocked, anticancer immunity is also blocked.

TGF-β exhibits various cellular functions, including cell proliferation, cell differentiation, and extracellular matrix restoration. TGF-β1 is a polypeptide of the TGF-β superfamily [24]. It has a significant preventing effect on immune system by blocking the expression of major histocompatibility complex (MHC) class II in cancer cells and deactivating tumor-infiltrating lymphocytes (TILs) and natural killer (NK) cells [25].

VEGF and TGF-β1 have opposite effects on endothelial cells. TGF-β1 promotes apoptosis, whereas VEGF inhibits apoptosis. TGF-β1 promotes the expression of FGF2, which in turn upregulates the synthesis of VEGF [26,27]. Flk-1 inhibits VEGF signaling, abolishing the apoptotic property of TGF-β1 and activation of p38 MAPK. Therefore, deactivation of p38 MAPK can block TGF-β1-induced apoptosis [28]. VEGF activates p38 MAPK in the absence of TGF-β1, which in turn promotes cell survival. Therefore, cross-talk between VEGF and TGF-β1 signaling converts VEGF/Flk-1-activated p38 MAPK into a proapoptotic signal, showing that VEGF can be converted into an apoptotic factor; thus, this mechanism represents a new method of antiangiogenesis treatment [29].

TNF is an extremely pleiotropic cytokine. It plays a dominant role in host defense, inflammation, and homeostasis. At the cellular level, it is involved in various processes, including apoptosis, angiogenesis, necrosis, immune cell activation, and cell differentiation and migration. The FDA has approved TNF-α as an adjuvant therapy for cancer, but chronic administration of TNF-α for cancer treatment is still debated. TNF-α promotes the release of angiogenic molecules, such as VEGFA and VEGFC. Studies have also showed that, in vascular endothelial cells, TNF-α enhances the transcription of the VEGFR2 gene. TNF-α also upregulates the transcription of the stimulatory protein (Sp)-1, which increases the binding of this factor to the Sp-1 binding site of the VEGFR2 promoter region, ultimately increasing the expression of VEGFR2 [6]. This indicates that the expression of the VEGF gene can be enhanced by TNF-α through the transcription factor Sp-1 [30]. The increased expression level of VEGF promotes angiogenesis, which in turn stimulates tumor progression.

**Figure 1:**
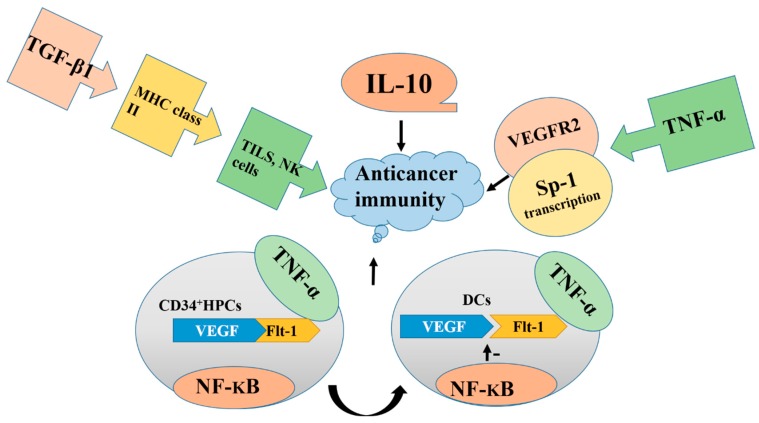
Overproduction of IL-10, TNF-α, TGF-β, NF-kB, and vascular endothelial growth factor (VEGF) blocks the functions of immune cells, deactivate TILs, and promotes tumor formation. Blocking the overproduction of these factors inhibits cancer progression. DCs, dendritic cells.

Interleukin 10 (IL-10), an immunosuppressive cytokine, is a regulatory element required for angiogenesis in many cancers. It facilitates the progression of various human cancers. The expression of IL-10 is markedly correlated with the expression of the VEGF121 gene and is responsible for its stimulation [31]. A study on the interaction between IL-10 and VEGF showed that IL-10 inhibits VEGF release in peripheral blood mononuclear cells, but its inhibitory effect has not been found in human trophoblasts [32]. Furthermore, production of macrophage-derived VEGF can be induced by both LPS/CGS21680 (generating the M1 phenotype) and PGE2 (generating the M2 phenotype). IL-10 suppresses the ability of the M1 phenotype to induce VEGF but has no effect on the M2 phenotype, which indicates that IL-10 affects only a certain type of macrophage-derived VEGF. Therefore, the effect of IL-10 on the regulation of macrophage-derived VEGF depends on the condition and phenotype of the macrophages [33] as described in Figure 1.

#### 3.1.2. VEGF and Receptor Tyrosine Kinases (RTKs)

Receptor tyrosine kinases are involved in cell growth and survival processes; they play a dominant role in angiogenesis and vasculogenesis and are critical to promote cancer neovasculature formation [33]. In various cancers, an elevated VEGFR level has been observed, which is correlated with metastasis and a poor diagnosis. Deregulation of receptor tyrosine kinases (RTKs) leads to the formation and progression of cancer. Thus, by blocking the signal of RTK, cancer formation can be prohibited. Various VEGFR inhibitors have been introduced to overcome the angiogenesis and lymphangiogenesis linked with tumor development [34].

The theory that VEGF is involved in tumor progression indicates that the signaling mechanism of VEGF is due to its interaction with some tumor-cell-expressed receptors. These include the RTKs VEGFR1, VEGFR2, and VEGFR3, which are known as Flt-1, Flk-1, and Flt-4, respectively [35]. Initially, the VEGF binding site was identified to be present on the surface of endothelial cells, but other evidence later reported indicates that the receptor of VEGF is also present in bone marrow cells [36]. These receptors exhibit overlapping, but the expression pattern is different: VEGFR2 in vascular endothelial cells [37], and VEGFR3 in lymphatic endothelial cells [38,39]. These receptors contain a domain that binds to a specific VEGF ligand. This ligand–receptor interaction is responsible for stimulating the tyrosine kinase domain of VEGFR. This stimulation induces an intracellular signaling transduction pathway involved in the regulation of cell proliferation, which ultimately leads to cancer progression [40]. Owing to hypoxia, the expression of VEGFR1 is upregulated via a hypoxia-inducible factor-1 (HIF-1)-dependent mechanism. VEGFR1 can bind with VGEFA, PLGF, and VEGFB. Recent studies on VEGFR1 showed that VEGFA, PLGF-2, and VEGFB increase sensitivity to cancer pain through activation of VEGFR1 in peripheral sensory neurons. Moreover, during β cell carcinogenesis, PLGF-1 reduces VEGFA-expression-dependent tumor angiogenesis [41]. Blockage of VGFR1 prevents cancer pain in diverse mouse models. Therefore, we can conclude from this finding that VGFR1 can promote cancer symptoms, and the development of a VGFR1 inhibitor can improve antiangiogenic cancer therapies [42].

**Figure 2:**
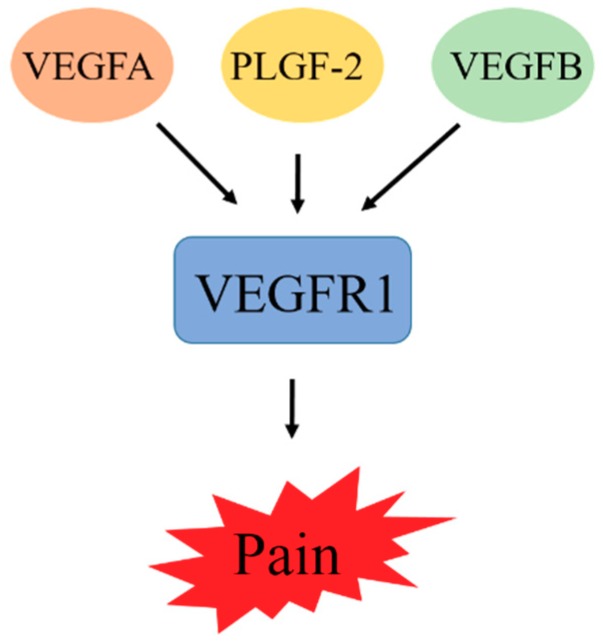
VEGFA, PLGF-2, and VEGFB increase sensitivity to cancer pain through activation of VEGFR1 in peripheral sensory neurons. Blockage of VGFR1 prevents cancer pain.

VEGFA plays the main role in angiogenesis by binding with two receptors: VEGFR1 and VGFR2. The juxtamembrane domain of VEGFR1 is involved in the suppression of kinase activity [43]. This suppression can be achieved through folding of the intracellular domain of VEGFR1 in such a way that checks the contact of regulatory sequences in the kinase domain. Supporting this concept, the tyrosine residue present in the kinase domain is not phosphorylated in the activated VEGFR1 as described in Figure 2. Therefore, in adulthood, the VEGFR1 tyrosine kinase is a positive regulator of macrophage function and it promotes inflammation, atherosclerosis, and cancer metastasis [44]. There are various tyrosine phosphorylation sites, and the proteins significantly related to these sites, including p85/phosphatidylinositol-3 kinase (PI3K) and growth factor receptor bound 2 (GRB2), have been studied in detail in various overexpression models [45]. However, the signaling pathway essential for VEGFR1 biology in vivo remains to be elucidated.

VEGFR1, compared with VEGFR2, has a higher affinity to VEGF, though tyrosine phosphorylation shows a weaker response to VEFGR1 than to VEGF [38]. This concept, along with the alternative splicing form of VEGFR1, shows that RTK acts as a decoy receptor by isolating VEGF from VEGFR2, thus affecting the regulation of VEGFR2 signaling. However, even in the absence of VEGFR2, VEGRF1 can be expressed by tumor cells, and it is likely to act as a signaling receptor and control major functions, but the exact mechanism is not clear. On the contrary, VEGFR3 is different from both VEGFR1 and VEGFR2 because it does not have to bind with VEGFA and acts as a receptor for both VEGFC and VEGFD in lymphangiogenesis [46]. Therefore, by inhibiting the RTK VEGFR1, cancer progression can be inhibited.

**Figure 3:**
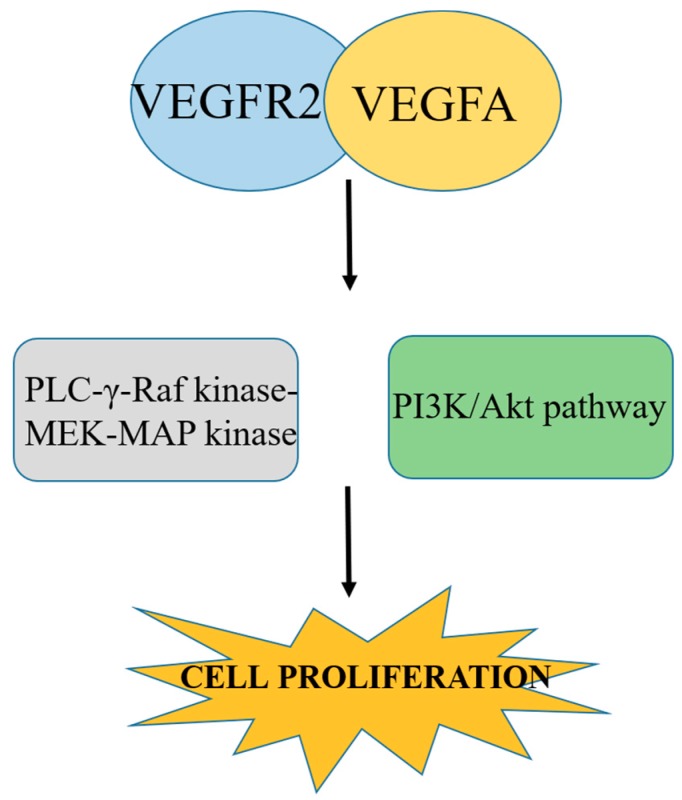
Binding of VEGFR2 with VEGFA causes autophosphorylation, which in turn causes activation of plc-γ-Raf kinase-MEK-MAP kinase and the PI3K/Akt pathway and promotes cell proliferation. Blocking this pathway inhibits cancer cell proliferation.

The RTK VEGFR2 is responsible for signaling in endothelial cells, which in turn is responsible for VEGF-controlled angiogenesis. VEGFR2 is also expressed by many tumor cells, but its role in tumor cells is independent on RTKs [47], which indicates that some other receptors are involved in the mediation of VEGF signaling. The binding of a VEGF ligand to its receptor depends on the presence of NRP1 and NRP2 because they are considered to be antiangiogenic targets. In comparison with VEGFR1, VEGFR2 has a lower affinity towards VEGF but higher affinity towards kinase activity. Binding of VEGFR2 with VEGFA mediates and stimulates a cascade of several signaling pathways, including the dimerization of receptor, which causes autophosphorylation of the tyrosine kinase domain. This autophosphorylation is responsible for the activation of PLC-γ-Raf kinase-MEK-MAP kinase and the PI3K/Akt pathway. This activation is responsible for cell proliferation of endothelial cell survival [48] as described in Figure 3. Therefore, blocking or inhibiting these pathways can inhibit cell proliferation, and ultimately be used to develop anticancer therapies.

#### 3.1.3. VEGF Interaction with NRPs

NRPs are non-tyrosine kinase receptors that bind with VEGF. It is well-known that tumor cells appear to lack some of the VEGF RTKs and response to other receptors involved in VEGF signaling. Recently, NRPs have gained interest as a VEGF receptor involved in the initiation and progression of cancer [49]. Initially, NPRs were identified as neuronal receptors for class 3 semaphorins, which have a role in the development of the nervous system [50]. NRPs cannot transmit an intracellular signal alone; thus, they require some co-receptor complexes. The two NPRs expressed in vertebrates, namely NRP1 and NRP2, are transmembrane glycoproteins exhibiting 40% homology at the amino acid level [51]. They consist of four different extracellular domains that promote ligand binding. They are also comprised of a short cytoplasmic domain that has no catalytic activity. Owing to the alternative splicing of NRP1 and NRP2, multiple isoforms can be produced [52]. NPRs can be modified through O-linked glycosylation, which can increase ligand binding and receptor expression [53,54].

**Figure 4:**
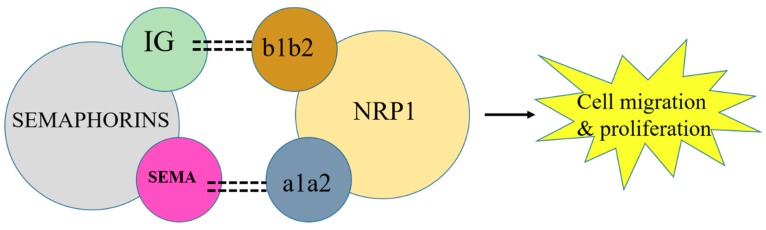
NRP1 in combination with semaphorin activates VEGF to initiate cancer cell migration. Blockage of NRP1 can inhibit tumor progression.

Class 3 semaphorins act as a bridge between the co-receptor complex plexin and NRP1 [55]. Semaphorins bind with NRP1 by their immunoglobulin (IG) and Sema domains. This immunoglobulin domain binds with the b1b2 domain of NRP1, whereas the Sema domain binds with the a1a2 domain of NRP1 [56,57]. However, the exact binding mechanism of semaphorin, NRP1, and plexin is not clear. Consequently, NRPs in cancer cells act as a VEGF receptor, which has led to the initiation of studies on the role of NRPs in tumor progression. Therefore, NRPs in combination with plexin form a complex that acts as a semaphorin receptor and is involved in cancer cell function as described in Figure 4. However, the role of plexin is yet to be elucidated properly in tumor cells. Owing to signaling through NRP1, VEGF initiates endothelial cell migration and adhesion, which are important in the early stages of angiogenesis.

In combination with integrins, NRPs are involved in cancer development. For example, NRP2 interacts with the α6β1 integrin, which in turn binds with the ligand laminin and causes the formation of focal adhesion kinases (FAKs) [58,59]. FAK, an important mediator of growth factor signaling, is involved in cell adhesion, migration, proliferation, and survival. FAK activity is altered to exert its role in tumor formation [60]. VEGF/NRP2 in combination with α6β1 facilitates the FAK-RAS signaling pathway activation that contributes to tumor de-differentiation and initiation [61]. This activation causes the activation of GLI1, a nuclear mediator of the Hedgehog pathway that mediates and regulates various genes important in different stages of cancer development and growth [62]. GLI1 activation promotes the expression of β cell-specific Molony murine leukemia virus integrations site 1 (BMI1). Many studies have proved that BMI1, a stem cell factor, is upregulated in various cancer cells [63]. Ultimately, all these factors cause cancer de-differentiation and initiation of cancer development.

**Figure 5:**
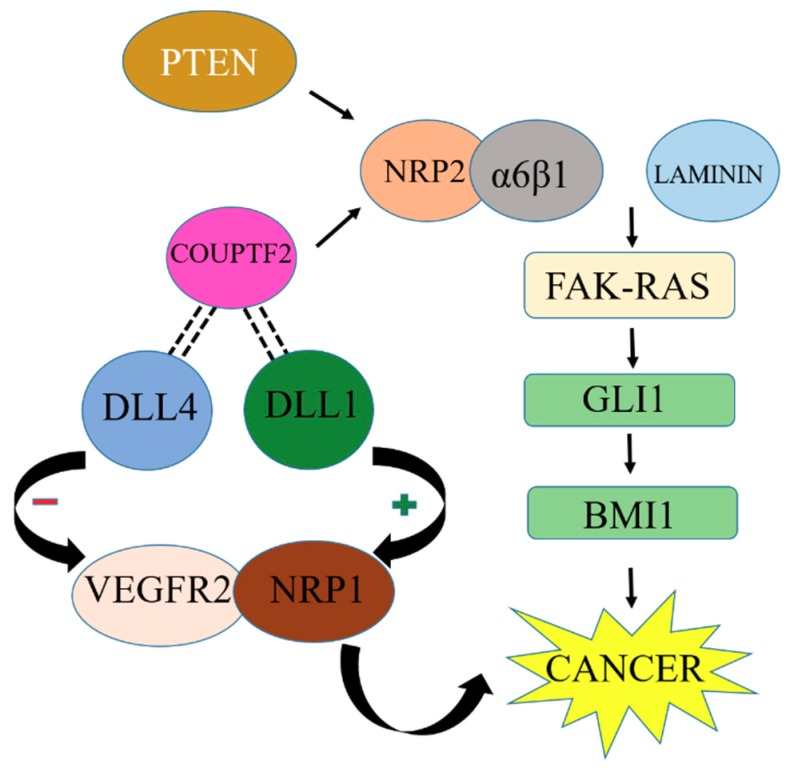
NRP2 interlinked with other cancer-promoting factors upregulates BMI1 and promotes cancer growth. Inhibition of BMI1 upregulation can stop cancer development.

In prostate cancer, phosphatase and tension homolog (PTEN) is responsible for the induction of NRP2 through the JUN N-terminal kinase (JNK)-JUN pathway. This pathway links the loss of tumor suppressor and induction of NRP2 transcription [64]. NRP2 transcription can also be stimulated by the COUPTF2 transcription factor in prostate cancer [65]. COUPTF2 also has an interesting role in the suppression of Notch ligand Delta-like 4 (DLL4). DLL4 can repress VEGFR2 and NRP1, although another ligand, DLL1, may be involved in the stimulation of VEGFR2 and NRP1 expression [66] as described in Figure 5. However, more studies are needed to explore the mechanism and components of VEGF signaling in cancer cells.

During the early stages of cancer development, cancer stem cells are present in the perivascular niche, which is located near endothelial cells. The self-renewal potential of cancer stem cells and their size are reduced through blockage of VEGFR2. Decreases in microvascular density and the renewal ability of cancer stem cells occur when VEGFR is deleted conditionally. Furthermore, deletion of NRP1 inhibits the function of VEGFR in the self-renewal ability of cancer stem cells. Therefore, these findings prove the importance of autocrine VEGF signaling in promoting the self-renewal ability of cancer stem cells and their proliferation in connection with NRP1 and VEGFR2.

NRP2 also downregulates the WD repeat and FYVE domain containing 1 (WDFY1). WDFY1 plays an important role in maintaining significant endocytic activity in cancer cells. The endocytic activity promotes the oncogenic activation of various cell surface receptors and autophagy. It was revealed that either downregulation of NRP2 or upregulation of WDFY1 can be an effective way to promote cell death in metastatic cancer [67].

Regulation of autocrine VEGF signaling in cancer can occur during receptor trafficking, which may be responsible for intracellular VEGFR signaling. Autocrine NRP1-VEGFFR signaling in gliomas may be responsible for the activation of VEGFR2, which is localized in the cytoplasm, thereby emphasizing the importance of intercellular VEGFR signaling for researchers. The key function of NRPs is to promote the signaling of VEGFR and other growth factor receptors [66].

Formation of NRPs complexes with VEGF RTKs (VEFGR1 and VEGFR2) can increase affinity towards VEGF. Although NRPs can also affect the other receptors involved in the functioning of tumor cells, the involvement of VEGF receptors in tumor formation is crucial [68]. It has been cleared that NRPs are expressed in cancer cells and their expression leads to tumor formation. Therefore, by understanding the exact association of NRPs with semaphorin and VEGFR, a novel anticancer agent can be developed. However, a deeper understanding is needed to elucidate the effect of NRP1 and NRP2 on cancer cells.

#### 3.1.4. VEGF and Integrins

Integrins, heterodimeric transmembrane proteins, play a role in cell adhesion, migration, and proliferation. In cancer cells, VEGF receptor can interact with integrins and activate or enhance the signaling of integrins. In coordination with a growth factor receptor, integrins can modulate cell functions by binding with their respective ligand. α3β1 binds with VEGFA and inhibits cell adhesion. Α9β1 binds with VEGFC and VEFGD and plays its role in lymphangiogenesis [69]. In tumor cells, VEGF receptors also interact with integrin signaling. This signaling is mediated by VEGFR2, which activates the binding function of multiple integrins in both endothelial and tumor cells through a pathway known as PI3K/Akt. This mediation can be bidirectional because evidence has shown that the αvβ3 integrin binds with VEGFR2 to form a complex that induces an increase in RTK phosphorylation in response to VEGF. NRPs can also bind with integrin and increase their role in cancer cells. For example, in breast cancer, VEGFR-bound NRP2 interacts with the α6β1 integrin, thus inducing the α6β1 integrin to bind with the ligand laminin, be involved in the formation of focal adhesions, and lead to the formation of focal adhesion kinases (FAKs). FAK is an important mediator of growth factor signaling, cell migration, proliferation, and survival. In cancer cells, FAK activity is altered and involved in tumor formation [60]. VEGF/NRP2 in connection with α6β1 facilitates FAK activation, which contributes to tumor de-differentiation and initiation [61]. VEGFR2 interacts with α1β1 and α2β1 integrins and regulates lymphangiogenesis [70]. Although the interaction between integrins and VEGF is very complex, we can suggest that by controlling the VEGF signaling, several cascades of reaction, which are directly or indirectly involved in tumor progression, can be controlled.

#### 3.1.5. Interaction of VEGF with HIFs

HIFs, key mediators of cancer cells in hypoxic response, interact and regulate several hundred genes. Members of the HIF family are involved in controlling various metabolic, angiogenic, and cell cycles. HIF modulation and interaction through different several genes could provide a potential therapeutic approach to a variety of pathologies, including cancer [71,72]. HIF-1α, a chief target for cancer treatment, interacts with VEGF and stimulates the development of new blood vessels, which in turn provide a sufficient supply of oxygen to cancer cells for their progression [72]. Another study revealed that in breast cancer cells, p16, a tumor suppressor gene, interacts and binds with HIF-1α, which in turn may alter HIF-1α’s capability to transactivate VEGF expression [73]. A deep understanding and the control of HIF-1α and VEGF interaction could be beneficial to develop novel strategies for cancer therapy.

#### 3.1.6. Interaction of VEGF with Other Receptors

An emerging concept has been noted about the interaction of VEGF receptors with another growth receptor. Although the interaction of VEGF RTKs by NRPs has been explained, there are many other growth factor receptors that may interact with them, such as the formation of a complex with MET [74]. NRPs also interact with other receptors as co-receptors. NRP1 interacts with MET and increases the ability of HGF to stimulate pancreatic carcinoma cell invasion along with the proliferation and survival of gliomas [75]. The interaction between the extracellular domain of NRP1 and epidermal growth factor receptor (EGF) leads to the stimulation of tumor cells’ response to EGF and transforming growth factor-α (TGF-α), which may contribute to EGFR activation. TGFβ signaling can occur because of the interaction of NRP1 and NRP2 with TGFβ. NRPs can bind various specific growth factors, such as HGF, platelet-derived growth factor, VEGF, PLGF, and TGFβ, but the exact mechanism remains to be determined.

#### 3.1.7. Interaction of VEGF with Other Signaling Pathways

VEGF has been shown to interact with many other cancer-related signaling pathways and inhibit cancer promotion and development. Chan PH and coworkers described that, after treatment with ischemic conditions, VEGF promotes the ERK1/2 signaling pathway and stimulates apoptosis in cerebral endothelial cells [76]. PI3K/Akt/mTOR/p70S6K, an important signaling pathway involved in cancer progression and metastasis, causes S100A4-promoted cell viability and migration, and promotes the VEGF secretion level through downregulation of E-cadherin in colorectal cancer cells [77]. Another study revealed that proinflammatory cytokines, including IL-1, IL-6, IL-8, TNF-α, and TGF-β, interact with VEGF and increase its expression in alveolar epithelial cells [78]. Another study revealed that in gastric carcinoma, VEGF and matrix metalloproteinase-2 and -9 are correlated with each other to regulate tumor angiogenesis, growth, invasion, and metastasis [79,80]. In contrast, in A549 lung cancer cells, MMP2 alters the expression of VEGF through the αVβ3-integrin-controlled PI3K/Akt signaling pathway [81].

### 3.2. Regulation of VEGF and Its Related Factors by Phytochemicals

#### 3.2.1. Flavonoids

##### Amentoflavone

Amentoflavone is a bioactive bioflavonoid present in *Chrozophora senegalensis.* It inhibits VEGF expression by interacting with and inhibiting the production and activation of transcription factor-2, cyclic adenosine monophosphate response binding protein, TNF-α, IL-1β, IL-6, and NF-kB to prevent the metastasis of and angiogenesis in human breast cancer [82,83]. It also interacts with VEGF and inhibits its expression by preventing the phosphorylation of VEGFR1 and VEGFR2. Furthermore, it inhibits endothelial cell migration induced by VEGFA and PLGF-1 [84]. This indicates that *C. senegalensis* can be a useful source of bioactive constituents that provide protection against cancer.

##### Baicalein

*Scutellaria baicalensis* is a flowering plant belonging to the Lamiaceae family. Baicalein, a flavone derivative isolated from this plant, suppresses CD45 expression and pulmonary metastasis. It markedly reduces lung cancer cell proliferation and cancer growth, as well as prolongs cell survival. In addition, it suppresses the protein expression of VEGF and 12-lipoxygenase. Moreover, it reduces microvessel density, the mitotic index, and VEGF and FGFR2 expression, as well as increases RB-1 expression [85]. A recent study revealed that it exhibits an anti-angiogenic effect by reducing the activator protein-1expression (AP-1), promoting AP-1 degradation, and weakening the MMP2/9 expression in relation to VEGF in an inflammation microenvironment [86].

##### Delphinidin

*Punica granatum* is a member of the Lythraceae family. Its fruit is famous worldwide owing to its nutraceutical and functional benefits. Delphinidin is an anthocyanidin found in *P. granatum* and it exhibits potential pharmacological activities, including antitumor, anti-mutagenic, anti-inflammatory, and antioxidant activities. In lung cancer cells, it exerts anti-angiogenesis activity by suppressing CoCl2- and EGF-induced VEGF protein production, mRNA expression, HIF-1α expression, and HRE promoter activity [78].

##### Kumatakenin

*Syzygium aromaticum* is a member of the Myrtaceae family. The flower bud of this plant has a long history of use as a herbal medicine and spice. Kumatakenin, an *O*-methylated flavonol anticancer agent isolated from this plant, inhibits ovarian cancer by suppressing MCP-1 and RANTES expression. In addition, it inhibits the cancer-promoting factors, including VEGF, MMP9, MMP2, and IL-10, in macrophages stimulated by ovarian cancer cells [87].

##### Licoricidin

*Glycyrrhiza uralensis* is a member of the Fabaceae family. Licoricidin, an isoflavone-type ingredient of this plant, exhibits an anticancer effect by suppressing the expression of VEGFA, COX-2, iNOS, HIF-1α, CD31, CD45, lyve-1, VEGFR2, VEGFR3, VEGFRC, VCAM-1, ICAM, and MMP9 [88].

##### Luteolin

*Arachis hypogaea* is a member of the Fabaceae family. Its seed oil exhibits pectoral, emollient, demulcent, and aperient effects, and it is consumed mainly as a nutritive food. In folk medicine, it is known to exert aphrodisiac and anti-inflammatory activities. Luteolin, a bioactive flavone derivative present mainly in its shell, exerts anticancer activities. It provides effects against breast cancer through anti-angiogenesis mechanism by inhibiting VEGF production and its binding with the receptor. In addition, it also downregulates epithelial–mesenchymal transition markers and lowers metastatic activity. Furthermore, it suppresses apoptosis and receptor tyrosine kinase activity, as well as prevents incipient colonization of breast cancer [89]. Recent studies have revealed that it inhibits vasculogenic mimicry formation and angiogenesis through inhibiting VEGF expression dependent on Notch 1 expression [90].

##### Oroxin B

*Oroxylum indicum* is a flowering plant belonging to the Bignoniaceae family. Traditionally, this plant is used as food, a traditional medicine, and part of marriage rituals. Oroxin B, a flavone glycoside isolated from this plant, suppresses proliferation of liver cancer cells. Furthermore, it suppresses COX-2/VEGF and PTEN/PI3K/Akt signaling pathways [91].

##### Quercetin

*Fagopyrum esculentum* is a member of the Polygonaceae family and popularly used in a cooked form for treating anemic patients. Quercetin, a plant flavonol, is contained in many plants, including onions, broccoli, raspberries, apples, citrus, *Nelumbo nucifera*, and leafy greens. It inhibits VEGF expression via multiple signaling pathways, including interaction with NF-kB nuclear transcription protein and hypoxia. Another study investigated the interaction of quercetin with PlGF-1 and VEGFA, but no interaction was found [84]. Other studies have shown that it reduces tumor weight by targeting VEGFR2 through the Akt/mTOR/P70S6K signaling pathway [92,93]. These findings indicate that edible or medicinal plants containing quercetin can be helpful in fighting cancer.

##### Scutellarein

*Scutellaria lateriflora* belongs to the Lamiaceae mint family, and it is employed as a mild sedative and sleep promoter. Scutellarein, a flavone present in *S. lateriflora*, reduces oxidative stress and suppresses the development of lymphoma and liver cancer. It also suppresses the expression of MMP2, MMP9, HIF-1α, Flt-1, and VEGFA. It also induces apoptosis by promoting caspase-3 DFF-40-controlled nucleosomal degradation [94,95,96].

##### Wogonin

*Scutellaria baicalensis* belongs to the Lamiaceae family and has been used over the years in traditional Chinese medicine to cure and treat respiratory infection, inflammation, insomnia, hemorrhage, hypertension, dysentery, and diarrhea. Wogonin, an *O*-methylated flavone found in this plant, suppresses cell proliferation, G0/G1 sub-population, and cell invasion. It downregulates the expression of ER-α, VEGF, Akt, and Bcl2, upregulates the expression of p53 and Bax, increases caspase-3 cleavage, and promotes apoptosis [96,97].

#### 3.2.2. Phenol, Polyphenol, and Phenolic Acid Derivatives

##### Arctigenin

*Arctium lappa,* also known as greater burdock, belongs to the Asteraceae family. It is cultivated in gardens to be used as a vegetable. In folk medicine, its dried root is known to exhibit blood-purifying, diaphoretic, and diuretic properties. It is an important ingredient of Essiac tea, which is used for cancer recovery. Arctigenin, an active lignin constituent of this plant, exerts an anti-inflammatory property and has the potency to cure prostate cancer by downregulating the expression of VEGF, EGF, and bFGF, as well as upregulating the expression of Bax/Bcl-2 [98].

##### Catapol

*Rehmannia glutinosa* is a species in the Orobanchaceae family. Traditionally, it has been used to cure diabetes, uterine bleeding, and urinary incontinence, as well as to regulate menstrual flow. Catapol, an iridoid glucoside isolated from this plant, shows several pharmacological activities, including antitumor, anti-inflammatory, and antiapoptotic activities. It ameliorates cancer in colon tissue by inhibiting the expression of VEGF, VEGFR2, HIF-1α/b, FGF, IL-1β, -6, -8, COX-2, and iNOS [99].

##### Curcumin

*Curcuma longa* is classified as a member of the Zingiberaceae family. In many Asian cuisines, it is considered to be a key ingredient. Usually, *C. longa* is used in the form of rhizome powder. Curcumin, a diarylheptanoid of the dried rhizome of *C. longa,* has been considered to possess an anticancer effect. It suppresses gastric carcinoma proliferation by inducing apoptosis in tumor cells, activating immune cells to release cytokines, and suppressing the STAT3, VEGF, HIF-1α, and DEC1 signaling transduction pathways [100].

##### Garcinol

*Garcinia indica* is a member of the Clusiaceae family and has been used for industrial, pharmaceutical, and culinary purposes. Garcinol, a polyisoprenylated benzophenone isolated from *G. indica* fruit, can potentially inhibit the growth and proliferation of oral cancer cells by inhibiting the expression of COX-2 and NF-kB to promote apoptosis [101].

##### Neoalbaconol

*Albatrellus confluens* belongs to the Albatrellaceae family. Neoalbaconol, an active constituent isolated from this plant, suppresses VEGF-induced proliferation, migration, invasion, and capillary-like tube formation of human umbilical vascular endothelial cells (HUVECs). Moreover, it inhibits the activation of the VEGF receptor and downregulates the related signaling pathway. It blocks EGFR-controlled VEGF formation and inhibits cancer angiogenesis in breast cancer [102].

##### Oxyresveratrol

Oxyresveratrol, a polyhydroxylated stilbene, is present in a large amount in *Morus alba,* and it has notably been employed as a herbal medicine. It inhibits cancer growth and metastasis, microvessel density, and micro-lymphatic vessel density. In addition, it also inhibits VEGFR-3, VEGFC, and CD31, and provides protection against cancer growth [103].

##### Resveratrol

*Polygonum cuspidatum* is a member of the Polygonaceae family. Resveratrol, an active stilbene ingredient of this plant, is comprised of various pharmacological activities, including anticancer activity. Resveratrol and its derivative, acetyl resveratrol, suppress the cell growth metabolism of SKOV-3 aggregates. The inhibition of cell growth interlinks with the attenuation of VEGF secretion and NF-kB protein levels [104].

##### Rosmarinic Acid

Rosmarinic acid is a water-soluble phenolic compound present in many medicinal plants, such as *Salviae miltiorrhizae* and *Rosmarinus officinalis.* It inhibits VEGF expression by inhibiting the production of IL-8 [105]. A recent study has shown that rosmarinic acid prevents the secretion of VEGF, IL-6, TGF-β, TNF-α, and NF-kB in H22 tumor-bearing mice, proving its potential as an antitumor agent [106]. Rosmarinic acid was indicated to prevent cancer by inhibiting inflammation and angiogenesis mechanisms.

#### 3.2.3. Terpenoids, Steroids, Lignans, and Fatty Acids

##### 3-*O*-acetyloleanolic Acid

*Vigna sinensis* is an herbaceous legume in the Fabaceae family. 3-*O*-acetyloleanolic acid, a pentacyclic triterpenoid compound present in this plant, has the potency to inhibit angiogenesis and cancer. It reduces the expression of VEGFA, suppresses the expression of VEGFR1 and VEGFR2 and the phosphorylation of PI3K, FAK, Akt, and ERK1/2, and inhibits cancer-promoted lymphogenesis and sentinel lymph node metastasis [107].

##### Actin

*Cimicifuga foetida* is a member of the Ranunculaceae family. It notably blocks cancer growth by suppressing cancer cell proliferation and reducing the migration and motility of cancer cells. Its mechanism involves the suppression of the VEGFR1, pERK, pINK, and JNK/ERK pathways. In addition, in breast cancer, it downregulates the expression of VEGFR1 and CXCR4 [108].

##### Ginsenoside Rg3

*Panax ginseng* belongs to the Araliaceae family. Ginsenoside Rg3, an active triterpenoid saponin, exerts an antiangiogenic effect by downregulating the VEGF/P38/ERK signaling pathway and inhibits the tube formation and migration of progenitor cells. It inhibits ectopic endometrium growth by suppressing the VEGFR-controlled PI3K/Akt/mTOR signaling pathway, exhibiting its anti-angiogenesis and proapoptotic effects [109].

##### Artemisinin

Artemisinin, a sesquiterpene, is a well-known antimalarial agent extracted from *Artemisia annua*. A recent study proved that artemisinin could be used as an anticancer agent. It prevents cancer by regulating multiple pathways. It decreases the expression level of VEGF, NF-kB, VEGF-Flt-1, and KDR/Flk-1 receptors in endothelial cells, thereby promoting anticancer activity [110].

##### Artesunate

Artesunate is a semisynthetic derivative of artemisinin. It prevents cancer development by inhibiting angiogenesis in a dose-dependent manner in HUVECs. It reduces immunosuppression and provides anticancer immunity to fight cancer by reducing the level of TGF-β1 and IL-10 [111]. This indicates that artesunate can be a significant anticancer agent.

##### Astragaloside IV

*Astragalus membranaceus* is a member of the Leguminosae family, and its dried root is a folk remedy used to repair and regenerate injured tissues and organs. Moreover, it helps to relieve stress and stress-related diseases. Astragaloside IV, a major constituent of this plant, suppresses cancer cell proliferation and terminates tumor development. It has been indicated to downregulate the PCNA, Ki67, MMP2 and 9, and VEGF signaling pathways. In addition, it potentially inhibits the MAPK/ERK signaling pathway [112].

##### Capsicodendrin

*Cinnamosma macrocarpa* is a member of the Canellaceae family, and its major active dimeric drimane-type sesquiterpene is capsicodendrin. Capsicodendrin inhibits VEGFR2, downregulates Akt, and induces autophagy by increasing LC3 cleavage and expression of autophagy-related genes (Atg3), Atg5, and beclin 1. Capsicodendrin-mediated reductions in sub-intestinal vessel formation and intersegmental vessel sprouting suppress sprouting angiogenesis and, ultimately, cancer growth [113]. It inhibits the phosphorylation of VEGFR2, eNOS, and Akt to regulate endothelial progenitor cells.

##### Celastrol

*Tripterygium wilfordii* belongs to the Celastraceae family and has traditionally been used to cure psoriasis and rheumatoid arthritis. Celastrol, a triterpene bioactive constituent, has been shown to exhibit an antitumor cancer effect by reducing the proliferation and migration of prostate cancer cells [114]. In addition, it suppresses the expression of VEGF and TNF-α.

##### Eriocalyxin B

*Isodon eriocalyx* is a member of the Lamiaceae family, and it exerts several potential biological activities. Eriocalyxin B, an active diterpenoid isolated from this plant, exerts its anticancer activity by suppressing VEGF-promoted cell proliferation, tube formation, cell migration, and cell invasion. In addition, it promotes G1-phase cell cycle arrest, which is interlinked with suppression of CDK4 and cyclin D1, and ultimately blocks the expression of phosphorylated retinoblastoma protein. Furthermore, it interacts with ATP-binding sites and suppresses VEGF-promoted phosphorylation of VEGF receptor-2, exhibiting its anti-angiogenesis activity to cure cancer [115].

##### Ginsenoside Rd

*P. ginseng* is an important plant that grows in China, Korea, and Siberia. The root of this plant is valuable and orally consumed to improve Alzheimer’s disease, memory, concentration, thinking power, muscle damage, physical stamina, and work efficiency. Ginsenoside Rd, a potent triterpenoid ingredient of this plant, suppresses VEGF-induced migration, proliferation, and tube formation of HUVECs. In addition, it ameliorates VEGF-promoted sprouting of vessels and suppresses vascular formation. In human breast cancer cells, it also exerts an angiogenic effect with a potential anticancer effect [116].

##### Ilexgenin A

*Ilex hainanensis* is a member of the Aquifoliaceae family and a traditional remedy for curing inflammation, dyslipidemia, and hypertension. Ilexgenin A, its novel, active pentacyclic triterpenoid component, exerts antitumor activity and promotes cell cycle arrest. In HepG2 cells, it suppresses the inflammatory cytokines IL-6 and TNF-α, inhibits VEGF production and transcription, and inhibits the PA3K and STAT3 signaling pathways [117].

##### Lupeol

*Senegalia visco* is a perennial tree belonging to the family Fabaceae. Lupeol, a phytosterol, is a major constituent present in this plant and it suppresses the migration, viability, and morphogenesis of HUVECs. It also lowers TNF-α transcription, downregulates VEGFR2 signaling, and phosphorylates FAK, PCL, Akt, and Src. In addition, it also interferes with cancer angiogenesis and suppresses cancer growth [118].

##### Oridonin

*Rabdosia rubescens* is an herbaceous plant belonging to the Labiatae family. Traditionally, it has been used to treat prostate cancer. Oridonin, a terpene compound isolated from *R. rubescens*, is employed as a therapeutic drug and dietary supplement. Pharmacologically, it exerts anticancer activity, suppresses HUVEC proliferation, migration, invasion, and tube formation, and induces cell apoptosis. Furthermore, it downregulates VEGFA, VAGFR2, and VEGFR3 expression, and upregulates TP53 expression. With these mechanisms, it inhibits cancer growth and metastasis [119].

##### Pomolic Acid

*Euscaphis japonica* belongs to the Staphyleaceae family. Its fruit is used as a drug. This plant contains an active triterpenoid, named pomolic acid, that suppresses cancer cell proliferation. The mechanism of this effect in MCF-7 and HUVEC cells involves the suppression of EGF-promoted VEGF/HIF-1α expression. In addition, pomolic acid downregulates EGF-promoted p38-MAPK phosphorylation and mTOR [120].

##### Stigmasterol

*Gundelia tournefortii*, a member of the Asteraceae family, is famous for its valuable health benefits for diabetes, intestinal diseases, cancer, and epilepsy. Stigmasterol, an abundant phytosterol isolated from this plant, exhibits potential anticancer properties through the VEGF signaling pathway [121]. Stigmasterol inhibits angiogenesis through the VEGF signaling network. It reduces the phosphorylation form of FAK, PCL, SRC, and VEGFR2 and inhibits endothelial cell proliferation and migration as well as well capillary network formation through disruption of the TNF–VEGFR2 axis [122].

##### Tanshinone IIA

*Salvia miltiorrhiza* belongs to the Lamiaceae family. It is famous for the use of its root in traditional Chinese medicine. Tanshinone IIA, a major, bioactive diterpene quinone compound present in this plant, controls the angiogenesis functions in HUVECs and inhibits VEGF-induced migration and tube formation of human progenitor cells (EPCs) without causing cellular toxicity. A detailed mechanism study indicated that the PLC, Akt, and JNK signaling pathways are involved in controlling angiogenesis [123].

##### Thymoquinone

*Nigella sativa* is a flowering plant belonging to the Ranunculaceae family. Thymoquinone, a major monoterpene isolated from this plant, has exhibited effects against several different types of cancers in a number of preclinical studies. It attenuates tumorigenic signaling, including those controlled by EGF, FGF, VEGF, TGF-β, and various metastatic, angiogenic, and pro-mitogenic factors [124].

#### 3.2.4. Coumarins

##### Daphnetin

*Daphne odora* is a member of the Thymelaeaceae family. The stems and flowers exert several biological activities, including ophthalmic, depurative, anti-spasmodic, antiphlogistic, and anodyne activities. Daphnetin, a bioactive constituent of this plant, has been shown to exert different pharmacological effects, such as anti-inflammatory, anti-arthritic, and antitumor effects. It inhibits angiogenesis induced by TNF-α and VEGF by downregulating the IKKS/IkBα/NF-kB, Src/Fak/ERK1/2, and Akt signaling pathways. Taken together, the potential of daphnetin as a multi-target anticancer agent has been shown [125].

##### Esculetin

The root of *Cichorium intybus*, a member of the Asteraceae family, is used to protect against dropsy and liver and spleen diseases. Esculetin, a coumarin derivative, inhibits VEGF-induced proliferation and DNA synthesis, as well as downregulates MMP9 expression in HUVECs. Furthermore, esculetin reduces VEGFR2 phosphorylation and downregulates the ERK1/2 and eNOS/Akt signaling pathways, showing its potential role in inhibiting cancer progression [126].

##### Scopoletin

*Nicotiana glauca* is a member of the Solanaceae family. The leaves of this plant are used in hunting rituals by the Cahuilla Indians. Moreover, the leaves are also used to treat swollen glands, an inflamed throat, sores, boils, wounds, cuts, bruises, and swellings. It actively inhibits FGF2, VEGFA, and ERK1, and promotes the inhibition of cancer development [127].

#### 3.2.5. Aldehydes, Ketones, Quinones, and Anthraquinones

##### α-/β-Thujone

*Thuja occidentalis* is a member of the Cupressaceae family. α- and β-Thujone, compounds isolated from this plant, decrease cell viability, proliferation, and angiogenesis, as well as induce apoptosis in glioblastoma cells. They suppress the expression of angiogenic markers, such as VEGF, CD31, and Ang-4, and promote neoplasia regression [128].

##### Emodin

*Aloe vera,* a member of the Asphodelaceae family, is used as an alternative and folk medicine. Emodin, an active anthraquinone present in *A. vera,* inhibits cancer growth by suppressing the expression of MMP7, MMP9, VEGF, EMT, N-cadherin, β-catenin, and Snail. In addition, it inhibits the Wnt/β-catenin signaling pathway through downregulation of target genes, including c-Myc, Cyclin-D1, and TCF4 [129].

##### Plumbagin

*Plumbago europaea,* commonly known as leadwort, is a member of the Plumbaginaceae family. It is mainly found in Central Asia. Its root is vesicant, sialagogue, odontalgic, emetic, and acrid. Chewing its root is beneficial to cure toothache. Plumbagin, an active compound of this plant, exerts an anti-angiogenesis effect by downregulating the PI3K/Akt, VEGF/KDR, and angiopoietins/Tie2 pathways and factors related with cancer angiogenesis and tumor cells, including bFGF, ET-1, CTFG, and VEGFB [130].

#### 3.2.6. Alkaloids

##### Berberine

*Coptis chinensis* belongs to the Ranunculaceae family and is used as a folk remedy to cure several diseases. Berberine, an active constituent of this plant, exerts its role in inhibiting cancer through an antiangiogenic mechanism; it inhibits VEGF expression in HUVECs with little toxicity [121].

##### Sinomenine

*Sinomenium acutum* is a member of the family Menispermaceae and used as a folk remedy to cure various diseases. Sinomenine, an active alkaloid isolated from this plant, has been identified as a potential anticancer and anti-invasion agent. It induces S-phase arrest and blocks clone formation to prevent proliferation. In osteosarcoma, it markedly inhibits metastasis and invasion with little toxicity. The mechanisms involved in its invasion inhibition include the suppression of STAT3 and CXCR4 phosphorylation, as well as downregulation of VEGF, RANKL, MMP2, and MMP9 expression [131].

##### Capsaicin

Capsaicin, an alkaloid/amide derivative, is a dietary-cancer-preventing agent found in *Capsicum frutescens*. It protects against tumor development and progression through different signaling pathways. It prevents melanoma cell proliferation by inhibiting the expression of VEGF. It downregulates the activation of focal adhesion kinase. It increases the degradation of hypoxia-inducible factor-1α and ultimately reduces VEGF expression [132]. Therefore, capsaicin is a potential anticancer agent interlinked with VEGF and VEGF-promoting factors.

#### 3.2.7. Others

##### α-hydroxy Succinamic Acid

*Eugenia jambolana* fruit, commonly known as Jamun, is broadly found in the Asian sub-continent. It is also distributed in other areas of the world, such as Madagascar and South America. The fruit pulp contains malvidin-diglucoside, petunidin, delphinidin, α-hydroxy succinamic acid, and anthocyanin. Both its seed and fruit pulp have a long history of use as medicines. Several of their pharmacological activities have been studied, including antidiabetic, anticancer, antiviral, and antibacterial activities. In vitro studies indicated that an ethyl acetate extract of *E. jambolana* exerts cytotoxic activity and inhibits cell proliferation and tube formation. In vivo studies indicated that the plant lowers body weight, cell number, and VEGF secretion. All these findings indicate the potency of *E. jambolana* as an anti-angiogenesis and pro-apoptotic agent [133].

##### Lectin

*Praecitrullus fistulosus* is a tropical medicinal and vegetable plant belonging to the Cucurbitaceae family. Lectin, a glycoprotein isolated from the fruit sap of *P. fistulosus*, exhibits its potential role against cancer development by decreasing VEGF secretion and inhibiting the expression of MMP2 and MMP9. Interestingly, it does not exhibit any side effects in normal mice [134].

##### Odisolane

*Morus alba* fruit is commonly known as mulberry, which is an edible fruit possessing emetic, anthelmintic, expectorant, odontalgic, laxative, tonic, and sedative properties. Odisolane, a novel oxolane derivative identified from mulberry fruit, exerts an anti-angiogenesis effect for cancer treatment by suppressing the protein expression of VEGF, p-Akt, and p-ERK [135].

##### PRP-S16

*Phellinus ribis* belongs to the Hymenochaetaceae family. PRP-S16 is a sulfated polysaccharide, which, using the chlorosulfonic acid method, is derived by sulfation from a glucan obtained from *Phellinus ribis*. It inhibits the proliferation, migration, and tube formation of cancer, as well as reduces the expression of VEGF, VEGFR-1, VEGFR-2, Akt, and ERK1/2 to inhibit cancer growth [136].

##### Sulforaphane

Broccoli and cauliflower, members of the cruciferous family, are famous vegetables around the world. Recently, research has revealed that sulforaphane, a member of the isothiocyanate group, is produced from glucoraphanin hydrolysis through myrosinase and has a promising anticancer effect [137]. It inhibits cancer cell angiogenesis through inhibiting HIF-1α and VEGF expression as well as the migration of human colon cancer cells [138]. Recently, research has revealed that in hepatocellular carcinoma, it exerts its anti-angiogenesis effect though suppressing the STAT3/HIF-1α/VEGF signaling network [139]. Moreover, human clinical studies have proven that it has a chemopreventive effect and can be added as a dietary supplement to slow down cancer progression.

## 4. Discussion

VEGF is an important and key component in cancer development, and it can be correlated with cancer transformation. This review reveals that VEGF enhances and encourages the function of cancer cells and maintains their self-renewal ability as well as provides an understanding of the potential use of VEGF signaling as a therapeutic target against cancer. The signaling pathway of VEGF and its potential role in tumor cells interact with many cancer-causing factors, including immune cells, RTKs, NRPs, integrin, HIFs, receptors, and other cancer-related signaling pathways to affect and regulate the function of cancer cells [140,141]. This review reveals many findings about and prospects for anticancer therapies through different novel pathways, such as an anticancer immune therapy that inhibits overproduction of IL-10, TNF-α, TGF-β, NF-kβ, and VEGF. Cancer patients feel severe and constant pain due to the involvement of VEGFA, PLGF-2, and VEGFB. VEGFR1 blockers reduce sensitivity to pain and cancer progression. Another way to inhibit cancer cell proliferation is to block the activation of plc-γ-Raf kinase-MEK-MAP kinase and PI3K/Akt pathways by blocking the binding of VEGFR2 to VEGFA. Blockers for NRP1 interlinked with semaphorin can deactivate VEGF secretion and ultimately suppress cancer development and progression [142]. Furthermore, downregulation of NRP2 or upregulation of WDFY1 can promote cell death in metastatic cancer by controlling the signaling of VEGFRFs and maybe a potential target-based anticancer therapy [143]. In addition, NRP2 upregulators bound with VEGFR2 and integrins can inhibit BMI1 upregulation, which may lead to suppressed cancer development [143]. Therefore, anticancer therapies can be developed using many signaling pathways involved in cancer development, instead of using the common pathways involved in cancer growth, which is disadvantageous due to the complexity of cancer progression and its treatment. Thus, studies should be conducted to develop new cancer inhibitors or suppressors targeting VEGF as an anticancer target through different signaling pathways.

**Figure 6:**
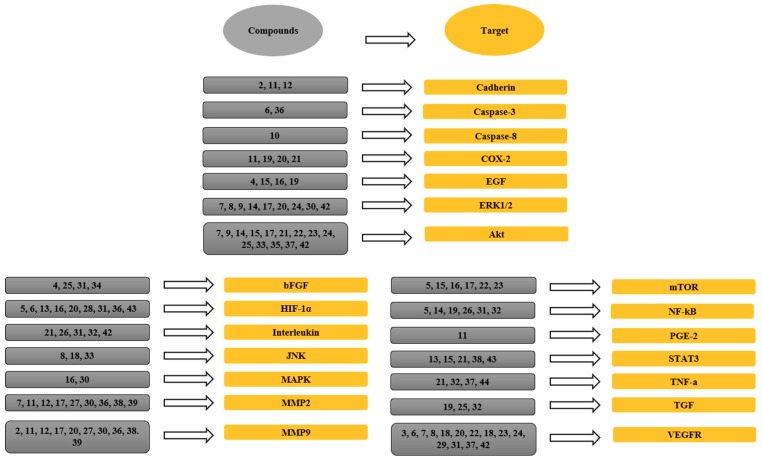
The links between phytochemicals and VEGF through different signaling targets. 1, Neoalbaconol; 2, Emodin; 3, Arctigenin; 4, Artemisinin; 5, Artesunate; 6, Esculetin; 7, Actein; 8, Berberine; 9, Curcumin; 10, Daphnetin; 11, Pomolic acid; 12, Quercetin; 13, Garcinol; 14, Licoricidin; 15, Ilexgenin A; 16, Eriocalyxin B; 17, Odisolane; 18, Oxyresveratrol; 19, Thymoquinone; 20, Scopoletin; 21, Oroxin B; 22, Ginsenoside Rd; 23, Ginsenoside Rg3; 24, PRP-S16; 25, Plumbagin; 26, Resveratrol; 27, Lectin; 28, Delphinidin; 29, Oridonin; 30, Astragaloside; 31, Catapol; 32, Rosmarinic acid; 33, Tanshinone IIA; 34, Baicalein; 35, Wogonin; 36, Scutellarein; 37, Lupeol; 38, Sinomenine; 39, Kumatakenin; 40, α-/β-Thujone; 41, Celastrol; 42, 3-*O*-Acetyl-oleanolic acid; 43, Sulforaphane; 44, Stigmasterol; 45, Luteolin.

This review also reveals the importance of natural products or their derivatives in cancer suppression or inhibition as a cheap and easily accessible source with a low number of side effects [13,14,15,16] as detailed in Figure 6. The combined use of synthetic drugs as chemotherapy, such as anti-metabolites, antibiotics, alkylating agents, microtubules inhibitors, monoclonal antibodies, and steroid hormones and their antagonists, causes patients to suffer from unbearable side effects, including resistance, multidrug resistance, toxicity, and treatment-induced tumor [144,145]. For example, alopecia, bone marrow suppression, stomatitis, and severe vomiting occur with all antineoplastic agents [145]. However, vomiting can be controlled by administration of an anti-emetic drug. Several toxic effects, such as myelosuppression, may cause infection. Other toxic effects include pulmonary fibrosis by bleomycin, cardiotoxicity by doxorubicin, and bladder toxicity by cyclophosphamide [145,146]. The duration of these side effects are very long; for example, alopecia is transient, but bladder, cardiac, and pulmonary toxicities are irreversible. To control these toxicities, patients need to take more medicine, which ultimately leads to a higher cost of therapy and reduced patient compliance. Therefore, there is a need to develop a medicine or drug with a multi-target property to overcome treatment toxicities. Natural products and their derivatives have the ability to act on different targets at the same time, which is why they are considered effective, cheap, and safe.

In addition, every plant is composed of unique phytochemicals that make them valuable to use under certain disease conditions or to act through different signaling pathways. Therefore, the main reasons for writing this review are to gather information about those plants and their distinct phytochemicals whose anticancer effect specifically targets VEGF and VEGF-interlinked cancer-promoting factors and has been studied recently. For example, earlier it was shown that sulforaphane affects different stages of cancer. Later, it was revealed that it suppresses hepatocellular carcinoma through suppressing the STAT3/HIF-1α/VEGF signaling network [139]. Stigmasterol was shown to exhibit antiangiogenic activity through inhibiting TNF-α. Recently, it was revealed that *Gundelia tournefortii*, a rich source of stigmasterol, affects angiogenesis through disruption of the TNF–VEGFR2 axis [122]. Therefore, the compelling data above reveal new insights and suggestions about the use of natural products and their derivatives against different oncogenic targets. For example, in ovarian and gastric cancer, emodin, esculetin, berberine, quercetin, licoricidin, lectin, astragaloside IV, scutellarein, sinomenine, and kumatakenin target metalloproteinases and inhibit VEGF to provide protection against these cancers. Ilexgenin A, rosmarinic acid, and lupeol target proinflammatory cytokines, such as TNF-α, to inhibit VEGF and ultimately provide relief against the pain in cancer immunotherapy [87,88,112,134]. Artemisinin, daphnetin, garcinol, resveratrol, catapol, and rosmarinic acid control the inflammatory response in cancer by targeting the NF-kB pathway [99,101,104,109,125].

Apoptotic invasion is considered to be one of the hallmarks of human cancers. Artesunate and scutellarein, members of the caspase family, control the apoptosis process though VEGF and provide a powerful therapeutic platform for the development of potential anticancer therapies [94,95,96].

In many human cancers, the PI3K/Akt/mTOR pathway is activated through VEGF and plays an important role in regulating angiogenesis. Esculetin, cirsimaritin, daphnetin, quercetin, odisolane, oroxin, ginsenoside Rg3, PRP-S16, plumbagin, tanshinone IIA, wogonin, lupeol, and 3-*O*-Acetyl-oleanolic acid target Akt and act as anticancer agents [95,109,118,125,126,130].

**Figure 7:**
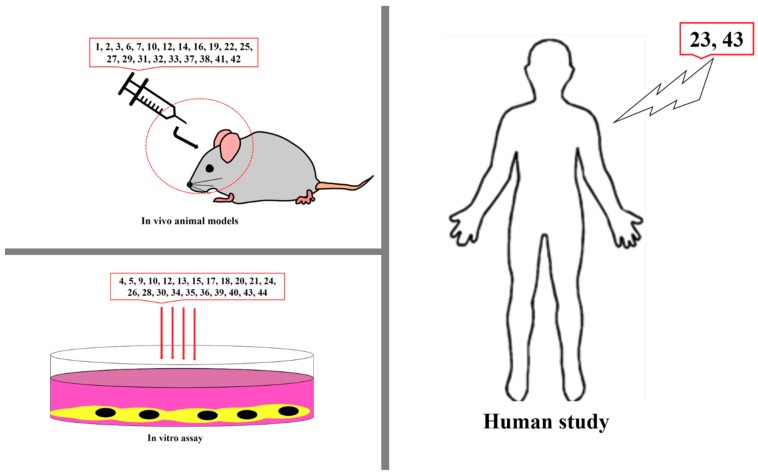
Studies on anti-VEGF chemicals in vitro, in vivo, and in humans. 1, Neoalbaconol; 2, Emodin; 3, Arctigenin; 4, Artemisinin; 5, Artesunate; 6, Esculetin; 7, Actein; 8, Berberine; 9, Curcumin; 10, Daphnetin; 11, Pomolic acid; 12, Quercetin; 13, Garcinol; 14, Licoricidin; 15, Ilexgenin A; 16, Eriocalyxin B; 17, Odisolane; 18, Oxyresveratrol; 19, Thymoquinone; 20, Scopoletin; 21, Oroxin B; 22, Ginsenoside Rd; 23, Ginsenoside Rg3; 24, PRP-S16; 25, Plumbagin; 26, Resveratrol; 27, Lectin; 28, Delphinidin; 29, Oridonin; 30, Astragaloside; 31, Catapol; 32, Rosmarinic acid; 33, Tanshinone IIA; 34, Baicalein; 35, Wogonin; 36, Scutellarein; 37, Lupeol; 38, Sinomenine; 39, Kumatakenin; 40, α-/β-Thujone; 41, Celastrol; 42, 3-*O*-Acetyl-oleanolic acid; 43, Sulforanphane, 44, Stigmasterol; 45, Luteolin.

However, ginsenoside Rg3, an active constituent isolated from *P. ginseng,* exhibits several pharmacological effects, including antioxidant, anti-inflammatory, immune-boosting, blood-sugar-lowering, and brain-cognition-improving effects. Moreover, this compound has been revealed to exert anticancer activity in humans by suppressing the PI3K/Akt and ERK1/2 signaling pathways correlated with VEGF and HIF-1α expression [109]. There are conflicting results from different clinical trials on its safety and efficacy as a complementary medicine or a dietary supplement [147,148,149]. Sulforaphane, an active constituent present in broccoli and cauliflower, exhibits a potent effect against several cancers, including cervical, bladder, prostate, and renal cell carcinoma, by hitting various signaling networks, such as the STAT3/HIF-1α/VEGF and Nrf2 signaling pathways [139]. Human clinical trials also proved that it has a chemopreventive effect and is safe, suggesting that it is a promising anticancer natural agent or dietary supplement [150,151]. All studies on the in vitro and in vivo effects of these natural products are shown in Figure 7. Moreover, the natural products mentioned in this review predominantly act on VEGF and its related promoting factors, but also exhibit other potential pharmacological effects that can be beneficial in controlling the side effects caused by the current use of clinical therapies (Table 1). Taken together, the data reveal the importance of this article as a source of information regarding the development of anticancer agents, and many future studies can be conducted to explore cancer therapies targeting the VEGF signaling pathway. Moreover, adding natural products into our daily diet can be helpful in fighting cancer progression and development.

## 5. Conclusions and Future Perspective

This review provides a deeper and up-to-date understanding of the effect of phytochemicals on VEGF interaction with multiple other cancer targets in tumor therapy. Blocking multiple targets in cancer is a promising strategy to develop effective cancer therapies. Therefore, this review suggests that a broad-spectrum approach to cancer therapy with phytochemical should be a feasible solution from a safety perspective. This new approach has the potential to be introduced to inhibit tumor cell function. In cancer cells, VEGF signaling interacts with VEGF RTKs, NRPs, integrin, and some other receptors, and can affect tumor cell functions and tumor initiation. Owing to this interaction, several signals are initiated that promote cancer cell progression, migration, adhesion, and proliferation. The development of novel therapeutic strategies can be made possible by considering and targeting the VEGF-linked promoting factors by using a single ingredient or a combination of therapeutic agents. Ilexgenin A, rosmarinic acid, and lupeol target proinflammatory cytokines, such as TNF-α, to inhibit VEGF and ultimately exert relieving effects against cancer-related pain. They may be interesting potential agents as a part of an anticancer therapy to relieve cancer-related pain.

## Figures and Tables

**Table 1:** Phytochemicals targeting VEGF as an anticancer therapy.

Sr. No	Plant Name	Active Constituent	Pharmacological Target	Biological in Cell/Organ	Pharmacological Activity	Reference
1.	*Albatrellus confluens*	Neoalbaconol	VEFGR/VEGF	Breast cancer xenograft model	Anticancer Anti-inflammatory Anti-rheumatoid Anti-proliferative Immunomodulating Hepatoprotective Anti-allergy Hypoglycemic	[102,152]
2.	*Aloe vera*	Emodin	MMP7, MMP9 VEGF, Wnt/b-catenin E-cadherin N-cadherin TCF-4, cyclin-D1 c-Myc	Colorectal cancer cells, mouse xenograft model	Anticancer Wound-healing Antifungal Anti-inflammation Immune-modulation Soothing Moisturizing	[129]
3.	*Arctium lappa*	Arctigenin	Androgen Rc VEGF, EGF, bFGF Bax/Bcl-2	Prostate tumor cell growth	Anticancer Anti-inflammatory Blood-purifying Kidney-protecting Hypoglycemic Immune-boosting	[153,154]
4.	*Artemisia annua*	Artemisinin	NF-kB/HIF-1α/VEGF Wnt/β-catenin, AMPK, NF-kB, AP-1, CREBP, MYC/MAX, mTOR	Human umbilical vascular endothelial cells (HUVECs)	Anti-malaria Anticancer	[155]
		Artesunate	VEGF-A VEGFR1,2 HIF-1α Caspase-3	Myeloid leukemia K562 cells,	Anti-malaria Anticancer	[156]
5.	*Astragalus membranaceus*	Astragaloside IV	MM2, MMP9, proliferating cell nuclear antigen (PCNA), Ki67, VEGF, Mitogen-activated protein kinase/Extracellular regulated protein kinase (MAPK/ERK)		Anticancer against glioma Antiangiogenic Immunoregulatory Antihypertensive Antioxidative Anti-inflammatory	[112]
6.	*Brassica oleraceae*	Sulforaphane	VEGF, STAT3, HIF-1α	Human colon cancer cells, HUVECs	Anticancer Antiangiogenic Immunomodulation Antiaging Antioxidant Anti-inflammatory Anti-neuroinflammatory Anticonvulsant Neuroprotective Anti-apoptotic Antidiabetic	[139,157]
7.	*Capsicum frutescens*	Capsaicin	VEGF, Cyclin D1, P38 MAPK, KDR/Flk-1, FAK/Akt, eNOS		Chemopreventive Anticancer Antiangiogenic Anti-migratory	[132]
8.	*Chicorium intybus*	Esculetin	MMP2 VEGF VEGFR-2 ERK1/2 eNOS Akt	HUVECs	Antiangiogenic Anticancer Anti-dropsy Stomach-controlling Anti-liver-diseases	[126]
9.	*Chrozophora senegalensis*	Amentoflavone	VEGFR1 VEGFR2 PLGF-1 IL-1β, IL-6 NF-kB		Neovascularization inhibitory Antiangiogenic Anticancer Anti-migratory Antimicrobial	[84,158]
10.	*Cimicifuga foetida*	Actein	VEGFR1/pJNK/pERK CD34/Factor VIII CXCR4	Breast cancer	Anticancer Antiangiogenic Antidiarrhea Anti-pain Anti-fever Anti-pneumonia	[108]
11.	*Cinnamosma macrocarpa*	Capsicodendrin	VEGFR2 Akt Beclin 1		Antiproliferative Cytotoxic Cytostatic Alpha-glucosidase inhibitory Antiviral	[159]
12.	*Coptis chinensis*	Berberine	VEGF miR-101/COX-2/AP-1/PGE2	B16F-10 melanoma cells and induced capillary formation in C57BL/6 mice	Anti-neurokeratin Anticancer Anti-inflammation Blood-pressure-lowering Anti-atherosclerosis	[121]
13.	*Curcuma longa*	Curcumin	DEC-1 HIF-1α VEGF STAT3	HUVECs, VEGF overexpressing tumor model in mice	Anticancer Anti-inflammatory Bile acid secretion-promoting Stomach-enhancing Cold-relieving Skin-soreness-relieving	[156]
14.	*Daphne odora*	Daphnetin	IKKs/IkBα/NF-kB Src/FAK/ERK1,2 Akt	HUVECs, rat aortic ring (RAR)	Anticancer Anti-arthritic Anti-inflammatory Anodyne Antiphlogistic Antispasmodic Anti-ophthalmic disease	[125]
15.	*Eugenia jambolna*	*α-hydroxy succinamic acid*	VEGF	A549 cells and activity of HUVECs	Cytotoxic Proapoptotic Antiangiogenic	[133,160]
16.	*Euscaphis japonica*	Pomolic acid	EGF/HIF-1α/VEGF p38-MAPK/mTOR	Breast cancer cells (MCF-7 and MDA-MB-231) HUVECs	Antitumor Anti-kidney disease Anti-arthritic Anti-rheumatoid	[120]
17.	*Fagopyrum esculentum*	*Q*uercetin	MMP2, MMP9 VEGF PKM2/GLUT1/LDHA Akt/mTOR	Human prostate tumor	Antioxidant Anti-inflammatory Anticancer Anti-metastasis Anti-headache Anti-circulatory problems	[161]
18.	*Garcinia indica*	Garcinol	NK-kB COX-2 VEGF	Pancreatic adenocarcinoma cells	Anticancer Antiproliferative Pro-apoptotic Cell-cycle-regulatory Anti-obesity	[101]
19.	*Glycyrrhiza uralensis*	Licoricidin	CD31, HIF-1α, iNOS, COX-2, LYVE-1, MMP9, ICAM-1, VCAM1, VEGFA/C/R2/R3	4T1 murine mammary carcinoma cells DU145 human prostate cancer cells	Antitumor Anti-inflammatory Estrogen-like action Adrenocorticol hormone-like action Antiallergic Anti-hyperlipidemia Liver cell protective	[88]
20.	*Ilex hainanensis*	Ilexgenin A	TNF-α IL-6 VEGF STAT3/PI3K	HepG2 cells HUVECs	Anticancer Anti-inflammatory Anti-hypertensive Anti-dyslipidemia Antimicrobial Anti-urinary infection	[117]
21.	*Isodon eriocalyx*	Eriocalyxin B	VEGF Cyclin-D1/CDK4 VEGFR2	Human umbilical vein endothelial cells, Zebrafish embryos, Mouse 4T1 breast tumor model	Antitumor	[115]
22.	*Morus alba*	Odisolane	VEGF p-Akt p-ERK	HUVECs	Anticancer Anti-constipation Tonic effects Blood sugar, pressure-lowering Anti-fever	[135]
23.	*Morus alba*	Oxyresveratrol	CD-31 VEGFR3 VEGF-C	Murine H22 hepatocellular carcinoma	Anticancer Anti-constipation Tonic effects Blood-sugar- and pressure-lowering Anti-fever	[103]
24.	*Nigella sativa*	Thymoquinone	TGF-β VEGF, FGF, EGF	Xenograft human prostate cancer (PC3) model in mouse Umbilical vein endothelial cell	Anticancer Chemotherapy- sensitizing	[162]
25.	*Niicotiana glauca*	Scopoletin	ERK-1 VEGFA FGF2	HUVECs	Anticancer Antitumor vascularization Skin-treating (swelling, bruises, cuts, boils, etc.)	[163]
26.	*Oroxylum indicum*	Oroxin B	COX-2/VEGF PTEN/PI3K/Akt	Hepatoma cell line SMMC-772	Anticancer (liver tumor)	[164]
27.	*Panax ginseng*	Ginsenoside Rd	Akt/mTOR/p70S6K VEGF	HUVECs Human breast cancer (MDA-MB-231) cell xenografts	Anticancer Anti-inflammatory Antioxidant Brain enhancement (cognition, memoryconcentration) Immune-boosting Blood-sugar-lowering Antiallergy Antiasthmatic Anti-proliferative Antiangiogenic Neuroprotective Analgesic Vasorelaxation Antidiabetic Antiaging	[116]
		Ginsenoside Rg3	VEGFR2 PI3K/Akt/mTOR	Bone marrow stromal cells Patient with acute leukemia		[109,157,165]
28.	*Phellinus ribis*	PRP-S16	VEGF VEGFR1,2 Akt ERK 1/2	EA.hy926 endothelial cells	Anticancer Anti-inflammatory Immune-boosting Liver/stomach-strengthening	[136]
29.	*Plumbago europaea*	Plumbagin	PI3K-Akt VEGF/KDR Angiopoietins, Tie2 CTGF, ET-1, bFGF	Human colon carcinoma and prostate cancer xenograft mouse models HUVECs	Anticancer Antiangiogenic Antibacterial Toothache-relieving Emetic Sialagogue	[130]
30.	*Polygonum cuspidatum*	Resveratrol	VEGF NF-kB IL-8	Hepatocellular carcinoma cells	Anticancer Antioxidant Anti-aging Skin inflammation Anti-CV diseases Antidiabetic	[104]
31.	*Praecitrullus fistulosus*	Lectin (PfLP)	VEGF MMP2, MMP9	EAC-bearing mice	Anticancer Antiangiogenic	[134]
32.	*Punica granatum*	Delphinidin	VEGF CoCl2- HIF-1α HRE	A549 lung cancer cells	Antioxidant Anti-inflammatory Anticancer Menopausal-symptoms-relieving	[78]
33.	*Rabdosia rubescens*	Oridonin	VEGFA VEGFR2,3 TP53	MDA-MB-231 and 4T1 breast cancer cells	Antiangiogenic Anti-metastasis Anticancer (prostate cancer) Benign prostatic hyperplasia (BPH) (enlarged prostate)-relieving	[164]
34.	*Radix Astragali*	Astragaloside IV	MAPK/ERK MMP2, MMP9 VEGF	Colon cancer cells	Anti-tumorigenic Antioxidant Anti-inflammatory Immune-boosting Blood-pressure-lowering Liver protection Heart-strengthening	[166]
35.	*Rehmannia glutinosa*	Catapol	VEGF, VEGFR2 HIF-1α, bFGF IL-1β/IL-6/ IL-8 COX-2	HUVECs Rat models	Anticancer Anti-inflammatory Cell protection (liver, brain) Heart-strengthening Blood-sugar-lowering	[99]
36.	*Salvia miltiorrhiza*	Rosmarinic acid	IL-1β, IL-6 TNF-α, VEGF TGF-β NF-kB/p65	H22 tumor-bearing mice HUVECs	Antioxidant Anti-inflammatory Antiallergic Anticancer Brain activation	[106]
		Tanshinone IIA	VEGF PLC/Akt/JNK	Cultured human retinal pigment epithelial cells Human endothelial progenitor cells in vitro and in vivo	Anticancer Antiangiogenic Cell protection (liver, brain)	[123]
37.	*Scutellaria baicalensis*	Baicalein	12-lipoxygenase VEGF FGFR2	HUVECs	Antitumor Anti-inflammation Antioxidant Cognitive-enhancing Neuroprotection	[85]
38.	*Scutellaria baicalensis*	Wogonin	ER-a VEGF Bcl-2 Akt Bax, p53	HUVECs	Anticancer (ovarian cancer) Anti-inflammation Antidiarrhea Anti-dysentery Anti-insomnia	[96,97]
39.	*Scutellaria lateriflora*	Scutellarein	VEGFA Flt-1 HIF-1α MMP2, MMP9 Caspase-3 DFF-40	Human retinal endothelial cells	Anticancer Antiangiogenic Anti-inflammation Sedative Analgesic Liver/nerve-cell-protecting	[94,95,96]
40.	*Senegalia visco*	Lupeol	TNF-a VEGFR-2 Src/Akt/PCL/PAK	HUVECs Cholangiocarcinoma growth in mice	Antitumor (cholangiocarcinoma) Antiangiogenic Anti-inflammatory	[118]
41.	*Sinomenium acutum*	Sinomenine	CXCR4/STAT3 MMP2, MMP9 RANKL/VEGF	Human osteosarcoma cells HUVECs	Anti-inflammatory Anticancer Anti-hypersensitivity Antimicrobial (amoeba) Anti-fever Analgesic (joint pain) Anti-rheumatoid Muscle-relaxing	[131]
42.	*Syzygium aromaticum*	Kumatakenin	MCP-1, RANTES IL-10 MMP2, MMP9 VEGF	Ovarian cancer cells Macrophages	Anticancer Carminative Anthelmintic Anodyne (painkiller) Anti-toothache	[87]
43.	*Thuja occidentalis*	α-/β-Thujone	VEGF Ang-4 CD-31	Lung metastatic C57BL/6 mice	Anticancer (brain) Antiangiogenic Anti-bronchial catarrh Anti-cystitis Anti-psoriasis Anti-respiratory tract infections (bronchitis, cold sores)	[128]
44.	*Tripterygium wilfordii*	Celastrol	VEGF	Macrophages Corneal neovascularization in rats	Anticancer (glioma, breast, prostate, lung, stomach, and brain) Antioxidant Anti-inflammatory Anti-autoimmune disease (multiple-sclerosis, rheumatoid, and psoriasis) Anti-allergic	[166]
45.	*Vigna sinensis*	3-*O*-Acetyl-oleanolic acid	VEGFR1,2 PI3K, FAK, Akt ERK12	Oral cancer sentinel lymph node animal model Squamous cell carcinoma (SCCVII) cells Human lymphatic microvascular endothelial cells (HLMECs)	Antitumor Anti-skin infection Anti-epileptic Anti-pain (chest) Sedative (tachycardia)	[107]
